# Fine‐scale diversity of prey detected in humpback whale feces

**DOI:** 10.1002/ece3.9680

**Published:** 2022-12-28

**Authors:** Rhonda D. Reidy, Matthew A. Lemay, Katie G. Innes, Rute B. G. Clemente‐Carvalho, Carly Janusson, John F. Dower, Laura L. E. Cowen, Francis Juanes

**Affiliations:** ^1^ Department of Biology University of Victoria Victoria British Columbia Canada; ^2^ Hakai Institute Genomics Laboratory Quadra Island British Columbia Canada; ^3^ Department of Mathematics and Statistics University of Victoria Victoria British Columbia Canada

**Keywords:** British Columbia, DNA metabarcoding, fecal sampling, humpback whale

## Abstract

Predator diets are largely influenced by prey availability and abundance. Yet, in heterogenous marine environments, identifying the prey species consumed by diving mammals remains a fundamental challenge. For rorqual whales, the energetic constraints of prey engulfment require that the whales seek areas of high prey abundance and execute discrete lunge feeding events on patches of high‐density prey. Prey occurrences in feces should therefore provide meaningful insight into the dominant taxa in food patches selected by the animal. We investigated the prey consumed by humpback whales in three regions in southern British Columbia (BC), Canada, using opportunistic fecal sampling, microscopy, and DNA metabarcoding of 14 fecal samples. Fish including Pacific herring (*Clupea pallasii*), hake (*Merluccius productus*), and eulachon (*Thaleichthys pacificus*) were the most common fish species potentially targeted by humpback whales in two regions. The krill *Euphausia pacifica* was the most prevalent invertebrate DNA detected in all three regions, while sergestid and mysid shrimp may also be important. High DNA read abundances from walleye pollock (*Gadus chalcogrammus*) and sablefish (*Anoplopoma fimbria*) were also recovered in one sample each, suggesting that juveniles of these semi‐pelagic species may occasionally be targeted. In general, we observed heavily digested fecal material that drove substantial dissimilarities in taxonomic resolution between polymerase chain reaction‐based and morphological analyses of the feces. Pacific herring and walleye pollock were the only prey species confirmed by both methods. Our results highlight that molecular and visual analyses of fecal samples provide a complementary approach to diet analysis, with each method providing unique insight into prey diversity.

## Graphical abstract

We investigated the prey consumed by humpback whales in three heterogenous regions in southern British Columbia, Canada, using opportunistic faecal sampling, microscopy, and DNA metabarcoding of 14 faecal samples. Fish including Pacific herring (*Clupea pallasii*), hake (*Merluccius productus*), and eulachon (*Thaleichthys pacificus*) were the most common fish species potentially targeted by humpback whales in two regions. The krill Euphausia pacifica was the most prevalent invertebrate DNA detected in all three regions, while sergestid and mysid shrimp may also be important.

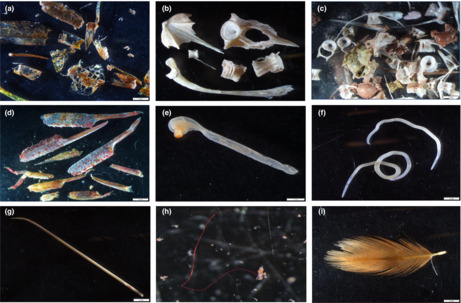

## 1 INTRODUCTION

Knowing what a predator eats is fundamental to understanding its habitat use and to modeling ecological systems (Young et al., 2015). Rorqual whales (Balaenopterid), such as blue whales (*Balaenoptera musculus*), fin whales (*B. physalus*), and humpback whales (*Megaptera novaeangliae*), are among the largest predators on earth and are major consumers in many ecosystems (Savoca et al., 2021). The extreme size of rorquals has been shown to scale with both their energy requirements (Guilpin et al., 2019; Potvin et al., 2012) and prey engulfment capacities (Kahane‐Rapport & Goldbogen, 2018). As diving predators, rorquals can adapt fine‐scale feeding tactics according to prey density, depth, and evasiveness to maximize their energy gain (Cade et al., 2016, 2020; Friedlaender et al., 2016, 2020).

During a foraging dive, rorqual whales routinely engulf and filter large mouthfuls of prey (Goldbogen, 2010; Goldbogen et al., 2017), wherein their foraging efficiency (energy gained vs. energy spent) is largely a function of prey encounter rate, prey type (energy content), and prey density (Goldbogen, 2010; Guilpin et al., 2019). However, complex decision‐making among foraging rorqual species likely occurs, particularly where prey patch densities may not always be optimal (i.e., prey intake per mouthful; Cade et al., 2021; Friedlaender et al., 2016, 2020). Although the consumptive abilities of rorquals are predicted to play an important role in pelagic food webs (Roman & McCarthy, 2010; Savoca et al., 2021), their prey selection in productive and variable nearshore habitats remains difficult to evaluate. Rorquals often forage well below the surface, precluding direct prey observations, and as a result, information on the full diversity of food items is lacking for many rorqual species.

Much of the progress in understanding rorqual foraging has come from the use of biologging tags for remote sampling of whale movement, often together with echosounders to draw inferences from the local prey‐scape (Cade et al., 2021; Friedlaender et al., 2009; Goldbogen et al., 2008; Hazen et al., 2009; Nowacek et al., 2011). By combining these three‐dimensional data with whale morphometrics, studies have also uncovered evolutionary differences in foraging patterns across rorqual species (Cade et al., 2016; Kahane‐Rapport et al., 2020; Kahane‐Rapport & Goldbogen, 2018). For instance, Kahane‐Rapport et al. (2020) showed that although blue whales have the largest mouths, and thus the largest prey engulfment capacities, they are constrained by proportionately longer water filtration times and may be the least maneuverable rorqual species (Cade et al., 2016; Kahane‐Rapport et al., 2020; Kahane‐Rapport & Goldbogen, 2018). In contrast, smaller humpback and minke whales (*B. acutorostrata*) exhibit shorter filtration times and greater maneuverability (Kahane‐Rapport et al., 2020). Both of these species typically feed in heterogenous nearshore habitats on both krill and agile fishes (Cade et al., 2016; Kahane‐Rapport et al., 2020; Kahane‐Rapport & Goldbogen, 2018).

Relatively little is known about the diversity of prey items consumed by these smaller and nimbler rorquals. For humpback whales in particular, while areas of high biological productivity have been reliable proxies for feeding habitat generally (Dalla Rosa et al., 2012), regional differences in diets and prey choices are suspected for humpback whale feeding groups throughout the North Pacific (Witteveen et al., 2009). Isotopic investigations using humpback skin and blubber samples have determined an overall general diet of fish and zooplankton for North Pacific humpback whales, with the animals in southern British Columbia (BC) and Washington State possibly feeding at a higher trophic level (i.e., more fish‐dominated diets) than in northern BC and southeast Alaska (Witteveen et al., 2011), presumably in relation to prey availability and abundance (Witteveen et al., 2008). In BC, prey items recovered from 287 stomachs of humpback whales harvested by commercial whaling from 1949 to 1965 identified euphausiids as the predominant prey (92% of stomachs; *Euphausia pacifica* and *Thysanoessa spinifera*), followed by copepods (4%) and fish (0.7%; Ford et al., 2009). However, these data were obtained prior to large climatic changes (regime shifts) in the 1970s and the late 1990s that affected production of major species groups in the North Pacific (Benson & Trites, 2002; Peterson & Schwing, 2003), and humpback whales are known to switch prey in response to climate oscillations (Fleming et al., 2016). More recent observations from surface‐feeding whales in BC indicate that euphausiids, abundant in shelf waters in southern BC (Phillips et al., 2022), continue to be a primary humpback whale prey item, but that schooling fish including Pacific sand lance (*Ammodytes personatus*), sardine (*Sardinops sagax caerulea*), and Pacific herring (*Clupea pallasii*) may be more prevalent in the diets of humpback whales in nearshore BC waters (Ford et al., 2009; McMillan, 2014).

Fecal samples provide a relatively noninvasive tool in dietary studies of marine mammals, as both prey DNA and prey hard parts (e.g., fish bones) can provide dietary insights (e.g., Jeanniard‐Du‐Dot et al., 2017; Thomas et al., 2017). DNA metabarcoding is now being applied to whale feces to characterize prey recently consumed (Carroll et al., 2019; de Vos et al., 2018; Ford et al., 2016), which can more accurately identify prey to the species level than traditional microscopic examinations of feces (Jeanniard‐Du‐Dot et al., 2017), and complement isotopic and fatty acid investigations that provide trophic‐level insights over the longer term (de Vos et al., 2018; Pompanon et al., 2012). One advantage of DNA metabarcoding is that it detects the presence of prey DNA in the whale's feces, even if indigestible hard parts were already excreted by the whale or degraded beyond visual identification (de Vos et al., 2018).

**FIGURE 1:**
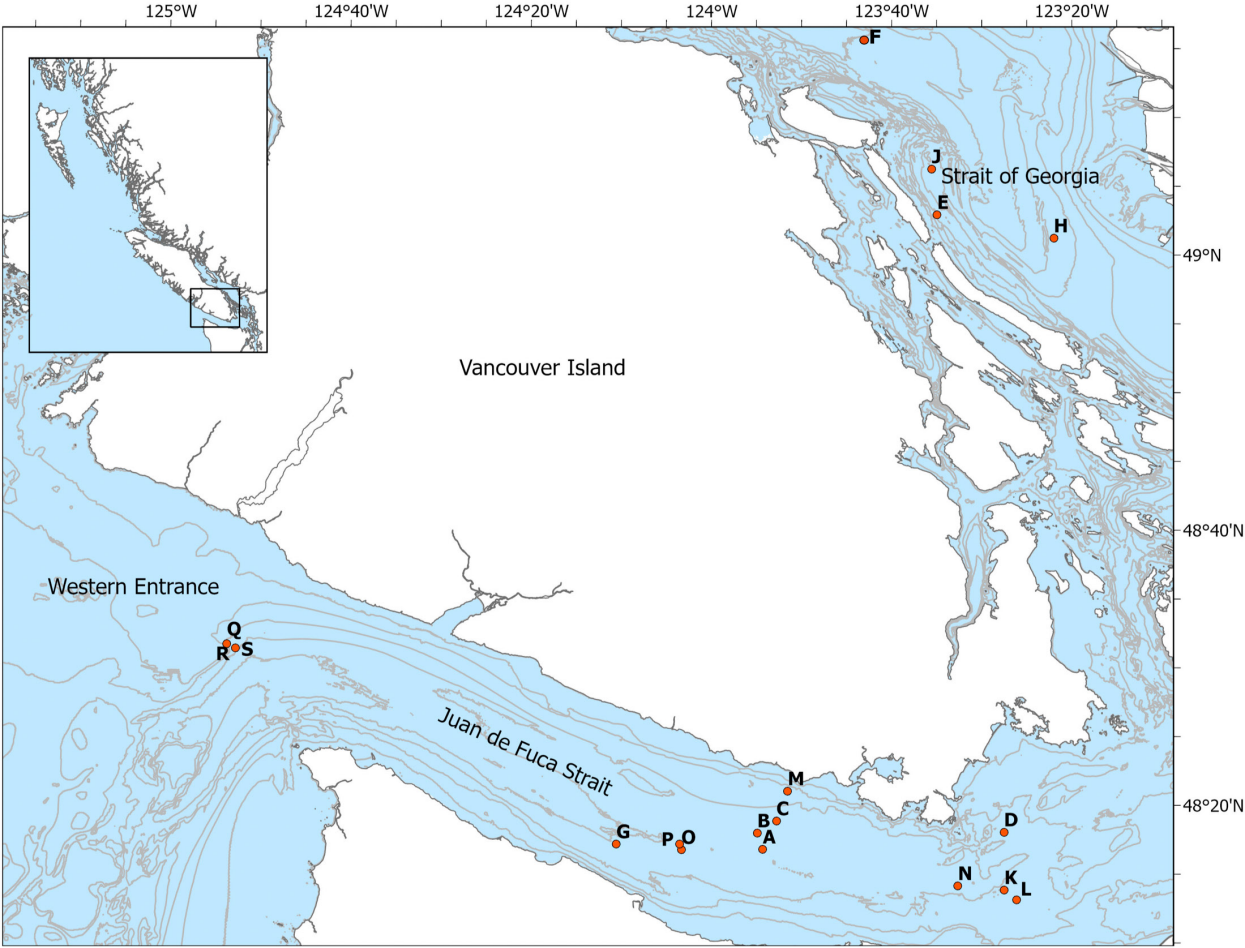
Map of southern Vancouver Island, BC, Canada, showing collection locations of humpback whale fecal samples in summer and fall of 2017–2020 from three geographic regions. Letters identify the individual fecal samples (*n* = 18)

We employed opportunistic fecal sampling and DNA metabarcoding to assess food items consumed by humpback whales foraging off southern Vancouver Island, BC. We assessed the molecular and morphological compositions of humpback feces collected over 4 years in summer through early fall in three biologically heterogenous regions: the Strait of Georgia, Juan de Fuca Strait, and the western entrance to Juan de Fuca Strait (Figure 1). Juan de Fuca Strait is a long, narrow, and turbulent tidal channel connecting the southern Strait of Georgia to the Pacific Ocean (Thomson, 1991). In spring, large volumes of freshwater are discharged into the Strait of Georgia from the Fraser River that flow persistently seaward above inflowing salty ocean water, strongly influencing the hydrodynamics of each of these regions and the marine food production of the entire system (Hickey & Banas, 2008; Perry et al., 2021; Thomson, 1991). Because humpback whales in BC demonstrate extremely strong site fidelity to feeding areas (Fisheries and Oceans Canada, 2013), we hypothesized that prey species detected in the feces would differ by sampling region. We also hypothesized that feces collected from humpback whales feeding at the western entrance to Juan de Fuca Strait would generate more diverse species results because this is an area of productive continental shelf waters (Hickey & Banas, 2008; Mackas et al., 1985; Thomson, 1991) and has consistently high numbers of feeding humpback whales (McMillan et al., 2022; Nichol et al., 2017). Our aim was to generate a qualitative baseline list of prey species in the humpback feces and to assess methodological challenges for improving future investigations.

## 2 METHODS

### 2.1 Study region and sample collection

A total of 18 fecal samples were collected from humpback whales during the summer and fall of 2017–2020 off southern Vancouver Island, BC, Canada (Figure 1). Collection of humpback whale fecal samples occurred in three regions: the Strait of Georgia (*n* = 4); Juan de Fuca Strait (*n* = 11); and slightly west of the western entrance to Juan de Fuca Strait (hereafter the ‘Western Entrance’; *n* = 3). Many of the samples (*n* = 13) were obtained opportunistically by commercial whale‐watching vessel crews operating within the waterways of Juan de Fuca Strait and the Strait of Georgia. Five fecal samples were obtained by us from Juan de Fuca Strait and the Western Entrance by close‐approach under Marine Mammal License MML‐45 and approved by the University of Victoria's Animal Care Committee under protocol 2017‐009. Most samples were collected within 1 m of the sea surface using a nylon pool skimmer net with 0.15 mm mesh size; however, four samples were obtained via a small plastic container or a bucket with a handle. All fecal samples were transferred into a new (i.e., clean) Ziploc bag and then double bagged for transport to the lab at the University of Victoria. A 20 ml subsample of each fecal sample was removed from the bag and preserved in 95% ethanol in a sterile screw cap collection tube for molecular analysis. A field negative control sample was collected with half of the samples (*n* = 9). These field controls consisted of a seawater sample collected at least 400 m from the fecal patch using a clean Ziploc bag, with a 20 ml subsample removed from the bag and preserved in sterile collection tubes in 95% ethanol. Each sample was labeled by the collector with the date, time, and GPS coordinates of sample collection, as well as the individual whale ID, and the behavior and number of whales present in the feeding group.

An additional lab control sample was obtained from the lab bench where the 20‐ml genetic subsamples had been aliquoted into sterile tubes. This sample was obtained by freezing deionized water from the lab in a clean Ziploc bag. The bag was filled to about a ¼ full, double bagged, and stored in the same freezer used to store the fecal samples. We processed the frozen water sample using the same bench space and protocols that we used to process the frozen fecal samples. We first wiped the working surface with chlorine bleach and used new gloves and a sterile scalpel blade for extracting a subsample from the frozen water. Fecal samples were stored at −80°C prior to subsampling, and at −20°C after preservation in ethanol until DNA analysis. The feces collected by commercial whale‐watching crews ranged in color from red to dark red/purple, were flocculent, and floated at the sea surface after the whale had moved away from the fecal patch. Samples were either delivered to us fresh on the day of collection or were stored frozen until we could retrieve them. One fecal sample collected by us was a purple‐brown liquid that sank rapidly within seconds of the whale defecating.

### 2.2 Microscopy

We identified the prey hard parts to the lowest possible taxon using a reference collection of prey species skeletons, a published taxonomic guide (Kozloff & Price, 1996), and consultation with local experts who specialize in marine invertebrates (L. Page, Department of Biology, University of Victoria, personal communication. October 2020) and zooplankton (M. Galbraith, Institute of Ocean Sciences, Fisheries and Oceans Canada, personal communication. April 2021). Frozen fecal samples were thawed and rinsed through a 0.5 mm sieve, and the remaining hard parts cleaned in distilled water. Bones and other material were examined under a dissecting microscope and initially assigned into two broad categories: prey (crustacean, gastropod, or fish) and non‐prey (e.g., worm, bivalve). Prey samples were further examined under a dissecting microscope and photographed using an Olympus stereoscope and cellSens imaging software. Prey items that were too digested for identification were preserved in 95% ethanol and excluded from the microscopic analysis. Quantitative proportions of the cleaned remains (i.e., number of individuals present) were not evaluated for two reasons. First, fecal collection was opportunistic with extremely small sample sizes in two of the three study regions. Second, the high level of digestion observed in most samples indicated that the hard parts would not likely provide unbiased, quantifiable estimates of numbers of prey consumed.

### 2.3 Molecular analysis

#### 2.3.1 DNA extraction

The fecal samples preserved in ethanol were homogenized using a vortex mixer for 10 s. We subsampled 1 ml of stool mixed with ethanol and transferred it to a clean Eppendorf tube (1.5 ml) for DNA extraction. The tubes were spun down for 1 min at 4500 *g*, and the ethanol was removed by pipetting without disturbing the stool pellet in the bottom of the tube. Immediately after removing the ethanol, 1 ml of InhibitEX buffer (Qiagen) was added to the stool sample. Total DNA was extracted from fecal samples using the QIAamp Fast DNA Stool Mini Kit (Qiagen) following the manufacturer's protocols. Control seawater samples were processed using an identical procedure as the fecal samples. An extraction blank (negative control) was processed at the same time as the biological samples to test for cross contamination during DNA extraction.

#### 2.3.2 Genetic library preparation

We used amplicon sequencing to quantify the diversity of fish and marine invertebrates in each fecal sample. We targeted fish using a short section of the 12S rRNA gene (*MiFish‐U*) described by Miya et al. (2015). We quantified marine invertebrates by targeting a short fragment of the Cytochrome c Oxidase subunit I gene (COI) using the primers *mlCOIintF* and *dgHCO2198* (Leray et al., 2013; Meyer, 2003). Library preparation was carried out using a two‐step polymerase chain reaction (PCR) approach based on Bourlat et al. (2016). The first PCR step was used to amplify the target regions and was carried out with three PCR replicates per sample, each with a 25 μl reaction volume containing 5.5 μl sterile Nuclease‐Free water, 0.6 μl of each primer (10 μM), 2 μl BSA (NEB), 12.5 μl 2× Taq (Froggabio), and 2 μl template. The thermal cycle settings were as follows: initial denaturation at 95°C for 5 min, followed by 40 cycles of denaturation at 95°C for 30 s, annealing at 63°C (12S rRNA) or 52°C (COI) for 30 s, and extension of 72°C for 45 s, with a final extension of 72°C for 5 min. We pooled the PCR replicates that corresponded to the same sample for each gene, and then performed a SPRI beads cleanup step (using 0.8 × beads) to remove excess primers and unspecific fragments below 300 bp. The SPRI beads cleanup followed the manufacturer's protocol, and samples were resuspended with 25 μl of sterile nuclease free water. For both genes, a negative control sample was included in all PCR reactions.

The second PCR step was used to attach Illumina adapters and unique barcodes to the amplicons for sequencing. This PCR was carried out with 25 μl reaction volume, containing 5 μl of sterile Nuclease‐Free water, 2.5 μl of each index (Nextera i7 and i5), 12.5 μl of 2× Taq (Froggabio), and 2.5 μl template (first PCR product). We used different combinations of indices (i5 and i7) to assign a unique identification to each sample. The second‐round thermal cycle settings were as follows: initial denaturation at 95°C for 3 min, eight cycles of denaturation at 95°C for 30 s, annealing at 55°C for 30 s, and extension of 72°C for 30 s, with a final extension of 72°C for 5 min. Another SPRI beads cleanup was carried out (using 0.8 × beads), and samples were resuspended with 25 μl of nuclease‐free water. We used the Qubit dsDNA High‐Sensitivity assay kit to quantify the DNA concentration of each sample, for posterior pooling of 40 ng of DNA per sample.

For the 12S rRNA library, an extra gel purification was necessary right after pooling the samples, when a 450 bp bacterial DNA fragment was also present in addition to the target fragment (~350 bp). We used the Wizard® SV Gel and PCR Clean‐UP System (Promega) to perform the gel purification and eluted the purified library with 50 μl of nuclease‐free water. The final concentration of each library pool was then estimated on a Qubit using the dsDNA High‐Sensitivity assay kit, and the BioAnalyzer trace obtained to confirm the fragment size of each library. Both libraries were sequenced using the Illumina MiSeq V2 chemistry (500 cycles) at the Hakai Institute genomics facility.

#### 2.3.3 Bioinformatics

We processed paired‐end, fastq‐formatted reads using the R package DADA2 (Callahan et al., 2016) and trimmed primers using the command‐line program cutadapt v2.10 (Martin, 2011). After learning error rates, dereplication, sample inference, and read merging with the default parameters in DADA2, we removed samples that had fewer than 50 true amplicon sequence variants (ASVs) present. Also, if ASVs were present in only a single sample with lower than 0.001 relative abundance, then that sample was removed (hereafter referred to as ‘singleton ASVs’). Bimeras (chimera sequences with two identifiable sources) were removed using the default parameters in DADA2.

Taxonomy assignment for both the COI and 12S libraries was carried out using BLASTN searches of the NCBI nucleotide database. We limited the BLAST output to hits that had ≥96% similarity to the query, ≥50% query coverage, and an *e*‐value of ≥10^−5^. All other parameters were set to the defaults of the program. We then used the Galaxy Tool LCA pipeline to determine the lowest common ancestor (LCA) taxonomy strings for each ASV (https://github.com/naturalis/galaxy‐tool‐lca). The top scoring blast hit was applied without using LCA if it had ≥98% similarity with the query, otherwise LCA was employed to determine a consensus taxonomy. The 12S data were then filtered to include only ASVs that were assigned to the two major classes of fishes: Actinopterygii (ray‐finned) and Chondrichthyes (cartilaginous). For the COI data, we removed ASVs that either lacked a phylum‐level assignment or did not annotate to a marine invertebrate (i.e., diatoms, algae, and vertebrates). We also removed all ASVs that were mis‐annotated to species that do not occur within the geographical range of this study. Reads annotating to the humpback whale were retained for both genes.

For fecal samples that had a corresponding field control (seawater sample), we subtracted the background DNA signal of ASV reads present in the seawater from the number of ASV reads present in the corresponding fecal sample. These seawater controls consisted of very small water samples (20 ml), and generally contained very little DNA (Appendix S1 Table A). We also subtracted the reads of any ASVs detected in the lab controls from the biological samples (i.e., lab bench control, DNA extraction control, and PCR controls). Following the subtraction of control reads from each ASV, we merged the sequence data from ASVs that annotated the same prey species.

#### 2.3.4 Identifying fish bones by DNA barcoding

During microscope examination of the feces, we found many tiny fish bones that could not be identified morphologically. To facilitate identification of these bone fragments, we used DNA barcoding to assign a species‐level identification. DNA extraction was carried out using the DNeasy Blood & Tissue Kit (QIAGEN) following the manufacturer's recommended protocols with the exception that samples were initially disrupted with stainless steel beads using a Tissuelyser II at 30 Hz for about 10–30 min, and the final elution was reduced to 50 μl Buffer AL.

We amplified the MiFish region of the 12S rRNA gene as described in Miya et al. (2015), using the primers *MiFish‐U‐F/R* (without Illumina overhangs) in a 25 μl reaction volume containing 1 μl of template DNA, 0.6 μl of each primer (10 μM), 3.75 μl of BSA, 12.5 μl of 2× Taq (Froggabio), and molecular grade water. The thermal cycle profile was as follows: initial polymerase activation at 95°C for 5 min, followed by 39 cycles of denaturation and amplification at 95°C for 30 s, 65°C for 30 s, 72°C for 45 s, and a final elongation step at 72°C for 5 min. The PCR amplification product was visualized on a 1.5% agarose gel stained with RedSafe Nucleic Acid Staining Solution (iNtRON Biotechnology). Nonpurified PCR product was submitted to the *Génome Québec Centre d'Expertise et de Services* (Montréal, Canada) for Sanger sequencing. BLASTN searches of the NCBI nucleotide database (nr/nt) enabled taxonomic assignment. We tested the accuracy of the results by including identified diagnostic bones of known species from Pacific herring and Northern anchovy.

## 3 RESULTS

Of the 18 humpback whale fecal samples, four contained no humpback whale DNA in the 12S data, and there were no prey items visible with microscopy. These samples represent failed attempts to collect the fecal sample and were subsequently omitted from further analysis. These included two samples from Juan de Fuca Strait and two from the Strait of Georgia. The analyses presented below are therefore restricted to the 14 successful samples.

### 3.1 Microscopy analyses

In most cases, the lowest identifiable taxonomic level for crustaceans was the order or class due to the advanced state of digestion of morphological features. Hard parts from 20 taxa belonging to nine phyla were morphologically identified for both invertebrates and fish combined. Peracarid and decapod shrimp were the most common prey identified by microscopy (Table 1). Of the identified shrimp remains, *Neomysis* spp. was the most common in six samples (43%), followed by Caridea (two samples), Cumacea (one sample), and Mysida (one sample, likely *Neomysis* spp.). No euphausiid (krill) hard parts were identified in the fecal samples; however, five samples contained partially digested crustacean fragments that were “euphausiid‐like”. These included all three samples from the Western Entrance, and one sample each from Juan de Fuca Strait and the Strait of Georgia.

**TABLE 1:** Prey taxa identified in the 14 fecal samples summarized by detection method and geographic region

Species	Strait of Georgia (*n* = 2)	Juan de Fuca Strait (*n* = 9)	Western Entrance (*n* = 3)	Total
Microscope				
Shrimp (*Neomysis* spp.)	1	5	0	6
Shrimp (Mysida)	0	1	0	1
Shrimp (Caridea)	0	2	0	2
Shrimp (Cumacea)	0	0	1	1
Pacific herring (*Clupea pallasii*)	0	2	1	3
Walleye pollock (*Gadus chalcogrammus*)	0	1	0	1
Northern anchovy (*Engraulis mordax*)	0	1	0	1
DNA				
Krill (*Euphausia pacifica*)	2	8	3	13
Krill (*Thysanoessa spinifera*)	1	6	2	9
Krill (*Thysanoessa raschii*)	0	6	0	6
Shrimp (*Sergestes similis*)	0	3	0	3
Shrimp (*Eualus avinus*)	0	2	1	3
Pacific herring (*Clupea pallasii*)	1	4	2	7
Pacific hake (*Merluccius productus*)	2	5	0	7
Eulachon (*Thaleichthys pacificus*)	0	6	0	6
Chinook salmon (*Onchorhynchus tshawytscha*)	1	4	0	5
Unknown rockfish (*Sebastes* spp.)	1	3	1	5
Coho salmon (*Onchorhynchus kisutch*)	1	3	0	4
Scalyhead sculpin (*Artedius harringtoni*)	1	3	0	4
California headlight (*Diaphus theta*)	0	4	0	4
High cockscomb (*Anoplarchus purpurescens*)	1	3	0	4

*Note*: Microscopy identified prey hard parts to the lowest possible taxonomic level. Prey detected by DNA were identified to species and a subset are summarized corresponding to prey taxa identified in ≥3 fecal samples. Values in the table correspond to the number of fecal samples that contained each prey item.

**FIGURE 2:**
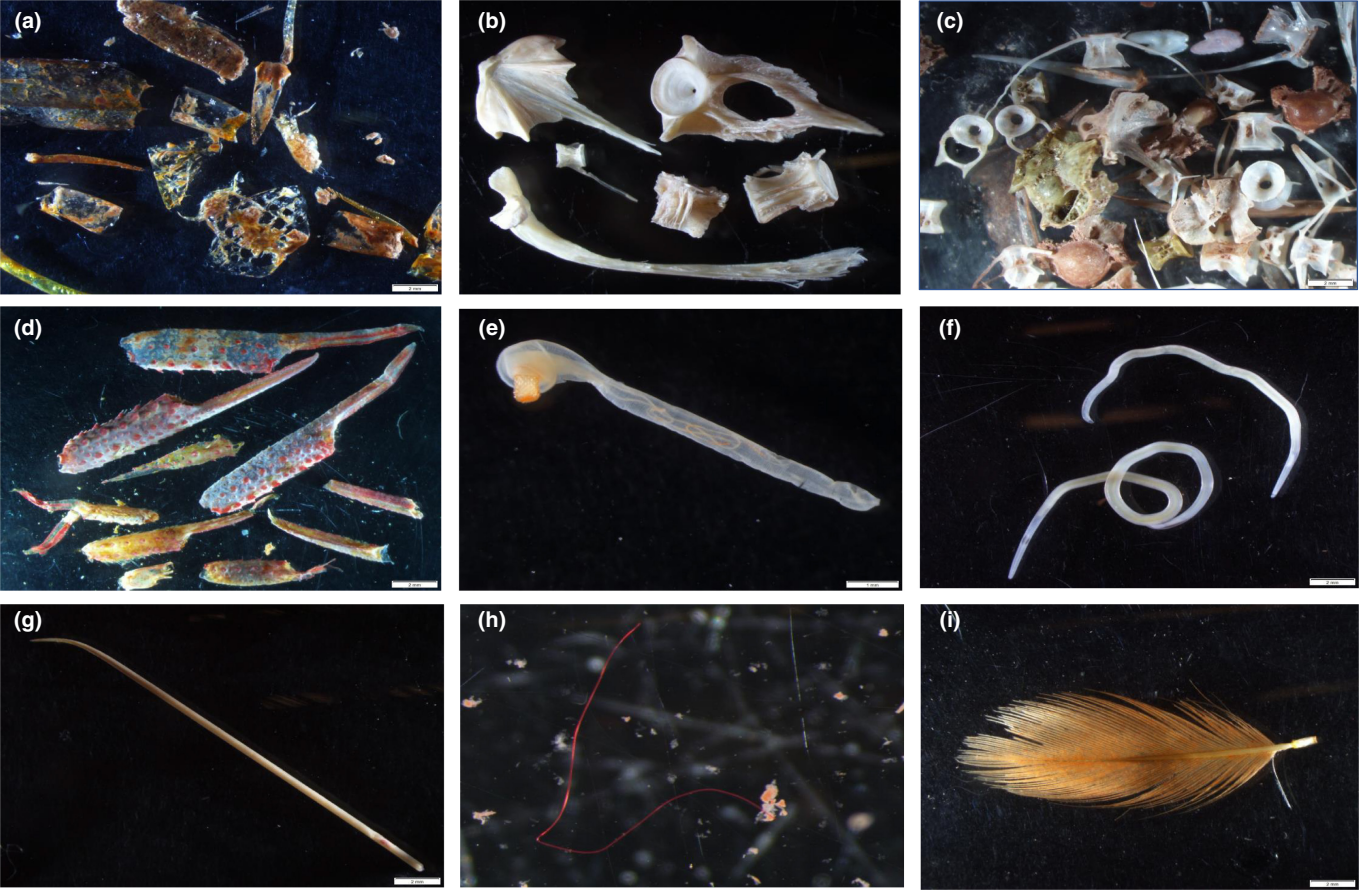
Example of hard parts in the humpback whale fecal samples identified by microscopy. (a) Pieces from two different shrimp (*Neomysis* spp. and Caridea); (b) cranial and vertebral bones of walleye Pollock; (c) bones from Pacific herring and northern anchovy from one sample; (d) claws of squat lobster (Galatheidae); (e) parasitic spiny‐headed worm (Acanthocephala); (f) parasitic worm (Nematoda); (g) whale baleen; (h) plastic fiber; (i) small feather. Scale bar = 2 mm

Bones from Pacific herring (*Clupea pallasii*) ranked second (three samples), followed by bones from walleye pollock (*Gadus chalcogrammus*; one sample). Northern anchovy (*Engraulis mordax*) bones were mixed in with one of the samples with herring bones (Figure 2). A linear regression comparing the standard length (SL) to the atlas width of two herring vertebra (c1 atlas) against a refence database (J. Qualley, unpublished data) suggests that the herring in the whale feces were 166 and 169 mm (176 and 179 mm fork length, respectively), which corresponds to age 2+ (Thompson et al., 2020).

Remains from nine taxa were not likely targeted prey items (e.g., worm, bivalve, and bryozoan). Crustacean fragments from crab (Brachyura and Anomura) and squat lobster (Galatheidae) were present in one sample from the Western Entrance (Figure 2). Mollusk fragments from a bottom‐dwelling snail, a cephalopod and bivalves were also present in this sample, as well as seafloor sediments, indicating that this whale had fed on or close to the seafloor. Other distinctly nonprey items identified in the samples included two parasitic helminths in five samples—Acanthocephala (spiny‐headed worm) and Nematoda (roundworm; Figure 2). Nematodes were identified in the three fecal samples from the Western Entrance, two of which also contained acanthocephalans. In two samples from Juan de Fuca Strait, one had only acanthocephalans present in the sample, while the other sample had only nematodes present. Both helminths occur in the digestive tract of large baleen whales, obtained by ingestion of infected crustaceans and fish (Hermosilla et al., 2015; Rice & Wolman, 1971). Strands of whale baleen and different colors of plastic fibers were also relatively common (five samples), and small bird feathers were present in two of the samples from the Western Entrance (Figure 2).

### 3.2 Molecular analyses

After bioinformatics processing 12S rRNA amplicon sequencing produced 7,590,343 reads (mean reads/sample = 291,936 from 316 ASVs), taxonomic filtering to retain only fish and humpback whale reduced these data to 7,430,672 reads (mean reads/sample = 285,795 from 267 ASVs) for downstream analyses. On average, 85% of the 12S rRNA gene sequences were attributed to the humpback whale (humpback whale sequence abundance ranged from 15.9% to 99.9% of samples). The most abundant and prevalent 12S prey ASVs were attributed to Pacific herring.

After bioinformatics processing COI amplicon sequencing produced 5,776,927 reads (mean reads/sample = 222,190 from 477 ASVs), taxonomic filtering of these data reduced it to 4,915,913 reads (mean reads/sample = 189,074 from 147 ASVs) for downstream analyses. Humpback whale represented an average of 99% of the COI reads per sample (humpback whale sequence abundance ranged from 93.8% to 99.9% of samples). The most abundant and prevalent prey ASVs were overwhelmingly from the krill, *Euphausia pacifica*.

**FIGURE 3:**
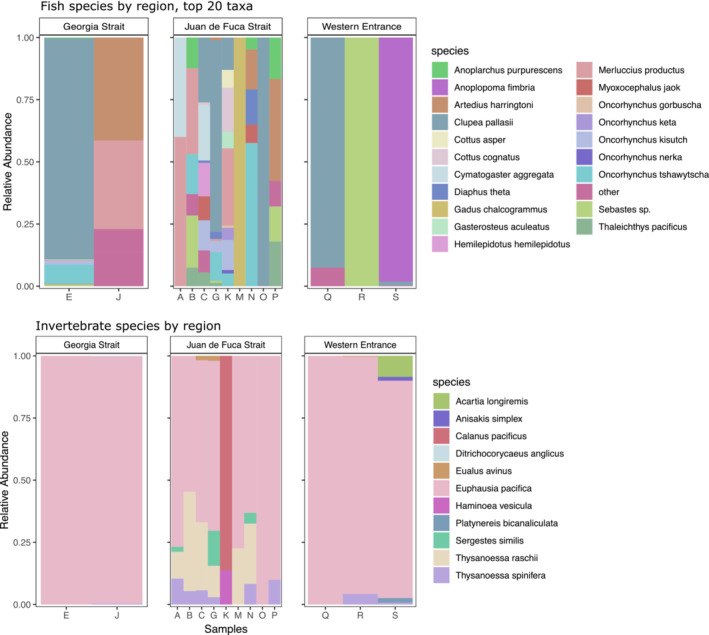
Visualization of the amplicon sequencing results per sample, shown separately for fish and invertebrates by geographic region, with the number of reads normalized to proportions. Each bar is a different fecal sample, identified by a letter in the order the sample was processed prior to amplicon sequencing. The colors show the relative proportion of prey species DNA reads in a sample based on presence (a low number of taxa per sample will show larger color blocks for the species present), but provide no quantitative information on read abundances. The top 20 most abundant taxa are presented for each gene, the remaining taxa are listed as ‘other’.

From the 14 samples retained, genetic analysis inferred 42 distinct species from 11 invertebrates and 31 fish (Figure 3). We considered a DNA detection in ≥3 samples (20%) to represent a “common” detection in this analysis. Krill dominated the invertebrate (COI) data, where sequences from *Euphausia pacifica* were the most common prey species detected in 13 of the 14 samples (93%), followed by *Thysanoessa spinifera* (nine of 14 samples; 64%) and *T. raschii* (six of 14 samples; 43%; Table 1). Two shrimp species, *Sergestes similis* and *Eualus avinus*, were detected in three samples each. From the fish data (12S), common detections included Pacific herring (*n* = 7; 50%), North Pacific hake (*Merluccius productus*, *n* = 7; 50%), eulachon (*Thaleichthys pacificus*, *n* = 6; 43%), Chinook salmon (*Oncorhynchus tshawytscha*, *n* = 5; 36%), rockfish (*Sebastes* sp., *n* = 5; 36%), coho salmon (*Oncorhynchus kisutch*, *n* = 4; 29%), scalyhead sculpin (*Artedius harringtoni*, *n* = 4; 29%), California headlightfish (*Diaphus theta, n* = 4; 29%), and high cockscomb (*Anoplarchus purpurescens*, *n* = 4; 29%; Table 1). All other species (invertebrate and fish) were detected in fewer than three samples. We generated ordination plots to look for patterns in the prey ASVs. However, there was not enough data for this analysis to be informative.

The highest abundances of fish sequences obtained in this study (all samples combined) came from Pacific herring, walleye pollock, Pacific hake, sablefish (*Anoplopoma fimbria*), slimy sculpin (*Cottus cognatus*), and coho salmon. However, despite the high total read count, walleye pollock and sablefish were only detected in one sample each, and thus were not considered a “common prey item” across samples. Conversely, the majority of sequences from Pacific hake were from a single sample; however, Pacific hake was detected at low read abundance in six additional samples and was classified a common prey item, despite its dominance in only one sample.

### 3.3 Regional patterns

#### 3.3.1 Strait of Georgia

The two samples from the Strait of Georgia (Samples E & J) comprised 78% of the *E. pacifica* sequence reads, suggesting that these two whales had consumed few other invertebrate prey items (Appendix S1 Tables B and C). One of these samples (sample J) also had DNA detections from the krill *T. spinifera* and the copepod *Ditrichocorycaeus anglicus*. However, neither sample had paired seawater from which to subtract a background signal of eDNA. Pacific herring was detected in sample E at relatively high sequence abundance. Nine other fish were detected in sample E at lower levels: Pacific hake, Chinook salmon, coho salmon, buffalo sculpin (*Enophrys bison*), rockfish (*Sebastes* sp.), high cockscomb, three‐spined stickleback (*Gasterosteus aculeatus*), tube‐snout (*Aulorhynchus flavidus*), and striped seaperch (*Embiotoca lateralis*). The other sample (sample J) had low read abundance for Pacific hake, scalyhead sculpin, prickly sculpin (*Cottus asper*), and ribbon prickleback (*Phytichthys chirus*). The mix of low‐abundance DNA from pelagic and nearshore species could indicate that these whales had recently fed close to shore, where incidental prey items were co‐ingested with targeted prey (e.g., euphausiids, Pacific herring). However, it could also represent DNA that was shed by these species into the seawater environment (eDNA) in which the feces were collected.

#### 3.3.2 Juan de Fuca Strait

Nine fecal samples were analyzed from the Juan de Fuca Strait region (Appendix S1 Tables B and C). Sample M was notable for being the only sample with walleye pollock, wherein walleye pollock completely dominated the sample (99.9% of fish sequences in this sample). The two other fish species (eulachon, California headlight fish) and two krill species (*E. pacifica*, *T. raschii*) also detected in this sample were all at low read abundances. One sample from Juan de Fuca Strait (sample K) had the greatest diversity of fish detected in a single fecal sample (13 species), and comprised >99% of the Pacific hake reads and 34% of the Pacific herring reads from this study. The wide mix of species in sample K could represent the heterogenous diets of ingested juvenile Pacific hake (Buckley & Livingston, 1997; Emmett & Krutzikowsky, 2008) and salmon (Daly et al., 2009; Duguid et al., 2021), and therefore secondary predation. Conversely, another sample from this region (sample A) had almost no fish DNA (total of 15 reads from two fish: Pacific hake, shiner perch), but a high relative abundance of *E. pacifica* (75% of the invertebrate reads for this sample). Notable was that fish bone fragments were also found in sample A, which DNA barcoding resolved as herring.

#### 3.3.3 Western entrance

Sablefish was the dominant prey item from one sample from this region (sample S; 98% of fish reads; Appendix S1 Tables B and C). Also detected in this sample was Pacific herring, padded sculpin (*Artedius fenestralis*), tube‐snout, and butter sole (*Isopsetta isolepis*; collectively <2% of fish sequences in the sample). In the two other samples from the Western Entrance, one (sample Q) had low detections of Pacific herring, padded sculpin, and striped seaperch. Herring bones were also recovered in this sample. The other sample (sample R) had only a very low detection from rockfish (26 reads) and no other fish DNA; however, it contained the seafloor sediments and hard parts from bottom‐dwelling invertebrate species. All three samples had invertebrate DNA detections. The sample with sablefish (sample S) contained DNA from two krill species (*E. pacifica*, *T. spinifera*), as well as one copepod (*Acartia longiremis*), polycheate (*Platynereis bicanaliculata*), and nematode (*Anisakis simplex*). The other two samples had DNA detected from *E. pacifica*; the sample with almost no fish DNA (sample R) had additional small detections from *T. spinifera* and the shrimp *Eualus avinus*.

### 3.4 Combining microscopy and molecular results

Prey detected by hard‐part microscopy and amplicon sequencing were combined to create a list of all detections in a presence‐absence matrix (Table 2). In total, 55 different organisms were identified across both methods. DNA detected twice as many items (42) as microscopy (20). Some of the partial classifications by microscopy (e.g., shrimp, copepod, euphausiid‐like pieces) were resolved to multiple different species by DNA. The major difference in results obtained from the two methods was due to the advanced state of digestion of the feces, as well as a higher detection sensitivity with DNA, particularly for rare species that were not likely targeted by the whale (e.g., sculpins). Likewise, krill were absent from the microscopy data, but were the most prevalent invertebrate group detected by DNA. Shrimp detections were the most variable between the two methods. DNA detected low reads from sergestid shrimp in three samples that were missed by microscopy, whereas mysid shrimp was identified in seven samples only by microscopy through expert analysis of diagnostic body parts. All but one sample that comprised mysid pieces had relatively low fish DNA detections; one mysid sample (sample J) with low fish detections had a high relative abundance of the krill *E. pacifica*. The sample with mysid pieces and high fish DNA detections contained the highest read abundances for Pacific hake and salmon in this study (sample K).

**TABLE 2:** List of taxa detected by microscopy and DNA obtained from 14 fecal samples

(A)					
Group	Common name	Order	Species	DNA	Microscope
Forage fish	Pacific herring	Clupeiformes	*Clupea pallasii*	7	3
Forage fish	Eulachon	Osmeriformes	*Thaleichthys pacificus*	6	0
Forage fish	Pacific sandlance	Perciformes	*Ammodytes personatus*	1	0
Forage fish	Northern anchovy	Clupeiformes	*Engraulis mordax*	0	1
Gadid	Pacific hake	Gadiformes	*Merluccius productus*	7	0
Gadid	Walleye pollock	Gadiformes	*Gadus chalcogrammus*	1	1
Gadid	Pacific grenadier	Gadiformes	*Coryphaenoides acrolepis*	1	0
Salmon	Chinook salmon	Salmoniformes	*Oncorhynchus tshawytscha*	5	0
Salmon	Coho salmon	Salmoniformes	*Oncorhynchus kisutch*	4	0
Salmon	Chum salmon	Salmoniformes	*Oncorhynchus keta*	1	0
Salmon	Pink salmon	Salmoniformes	*Oncorhynchus gorbuscha*	1	0
Salmon	Sockeye salmon	Salmoniformes	*Oncorhynchus nerka*	1	0
Perch	Shiner perch	Perciformes	*Cymatogaster aggregata*	2	0
Perch	Striped seaperch	Perciformes	*Embiotoca lateralis*	2	0
Perch	Kelp perch	Perciformes	*Brachyistius frenatus*	1	
Flatfish	Butter sole	Pleuronectiformes	*Isopsetta isolepis*	1	0
Sculpin	Scalyhead sculpin	Scorpaeniformes	*Artedius harringtoni*	4	0
Sculpin	Buffalo sculpin	Scorpaeniformes	*Enophrys bison*	2	0
Sculpin	Red Irish lord	Scorpaeniformes	*Hemilepidotus hemilepidotus*	2	0
Sculpin	Plain sculpin	Scorpaeniformes	*Myoxocephalus jaok*	2	0
Sculpin	Padded sculpin	Scorpaeniformes	*Artedius fenestralis*	2	0
Sculpin	Slimy sculpin	Scorpaeniformes	*Cottus cognatus*	2	0
Sculpin	Prickly sculpin	Scorpaeniformes	*Cottus asper*	1	0
Sculpin	Cabezon	Scorpaeniformes	*Scorpaenichthys marmoratus*	1	0
Other	Unknown rockfish	Scorpaeniformes	*Sebastes* sp.	5	0
Other	California headlight	Myctophiformes	*Diaphus theta*	4	0
Other	High cockscomb	Perciformes	*Anoplarchus purpurescens*	4	0
Other	Penpoint gunnel	Perciformes	*Apodichthys flavidus*	2	0
Other	Ribbon prickleback	Perciformes	*Phytichthys chirus*	2	0
Other	Sablefish	Scorpaeniformes	*Anoplopoma fimbria*	2	0
Other	Three‐spined stickleback	Gasterosteiformes	*Gasterosteus aculeatus*	2	0
Other	Tube‐snout	Gasterosteiformes	*Aulorhynchus flavidus*	2	0
Other	Unidentified bones				3

(B)									
Group	Initial ID	Phylum	Class	Order	Infraorder	Family	Species	DNA	Microscope
Crustacean	Krill	Arthropoda	Malacostraca	Euphausiacea		Euphausiidae	*Euphausia pacifica*	14	0
Crustacean	Krill	Arthropoda	Malacostraca	Euphausiacea		Euphausiidae	*Thysanoessa spinifera*	9	0
Crustacean	Krill	Arthropoda	Malacostraca	Euphausiacea		Euphausiidae	*Thysanoessa raschii*	6	0
Crustacean	Shrimp	Arthropoda	Malacostraca	Decapoda		Sergestidae	*Sergestes similis*	3	0
Crustacean	Shrimp	Arthropoda	Malacostraca	Mysida		Mysidae	*Neomysis* spp.	0	6
Crustacean	Shrimp	Arthropoda	Malacostraca	Mysida				0	1
Crustacean	Shrimp	Arthropoda	Malacostraca	Decapoda	Caridea	Thoridae	*Eualus avinus*	3	0
Crustacean	Shrimp	Arthropoda	Malacostraca	Decapoda	Caridea			0	2
Crustacean	Shrimp	Arthropoda	Malacostraca	Cumacea				0	1
Crustacean	True crab	Arthropoda	Malacostraca	Decapoda	Brachyura (megalopa)			0	1
Crustacean	True crab	Arthropoda	Malacostraca	Decapoda	Brachyura			0	1
Crustacean	Non‐true crab	Arthropoda	Malacostraca	Decapoda	Anomura			0	1
Crustacean	Squat lobster	Arthropoda	Malacostraca	Decapoda	Anomura (Galatheidae)			0	1
Crustacean	Decapod	Arthropoda	Malacostraca	Decapoda				0	1
Crustacean	Copepod	Arthropoda	Hexanauplia	Cyclopoida		Corycaeidae	*Ditrichocorycaeus anglicus*	1	0
Crustacean	Copepod	Arthropoda	Hexanauplia	Calanoida		Acartiidae	*Acartia longiremis*	1	0
Crustacean	Copepod	Arthropoda	Hexanauplia	Calanoida		Calanidae	*Calanus pacificus*	1	0
Crustacean	Copepod	Arthropoda	Hexanauplia	Calanoida		Candaciidae	*Candacia columbiae*	0	1
Crustacean	Hyperiid amphipod	Arthropoda	Malacostraca	Amphipoda				0	1
Crustacean	Ostracod	Arthropoda	Ostracoda					0	1
Crustacean	Unidentified pieces							0	5
Mollusk	Snail	Mullusca	Gastropoda	Cephalaspidae		Haminoeidae	*Haminoea vesicula*	1	0
Mollusk	Snail	Mullusca	Gastropoda					0	1
Mollusk	Cephalopod	Mullusca	Cephalopoda					0	1
Mollusk	Bivlave	Mullusca	Bivlavia					0	1
Other	Bryozoan	Bryozoa	Stenolaemata	Cyclostomatida				0	1
Other	Foraminifera	Retaria						0	1
Other	Hydromedusae	Cnidaria						0	1
Segmented worm	Worm	Annelida	Polycheata	Phyllodocida		Nereidiformia	*Platynereis bicanaliculata*	1	0
Segmented worm	Worm	Annelida	Polycheata					0	1
Endoparasite	Worm	Nematoda	Chromadorea	Rhabditida	Ascaridomorpha	Anisakidae	*Anisakis simplex*	1	0
Endoparasite	Worm	Nematoda						0	4
Endoparasite	Spiny‐headed worm	Acanthocephala						0	3
Organic	Baleen								5
Organic	Bird feather								2
Organic	Fur/hair (animal)								3
Organic	Plant material								4
Inorganic	Seafloor sediments								1
Inorganic	Plastic fibers								7

*Note*: Species detection is indicated by a value corresponding to the number of fecal samples that contained the prey item. A zero‐value indicates no detection by the analysis method. (A) Fish taxa were identified to genus and species. (B) Invertebrate taxa were identified to the lowest taxonomic level. Included in B are the non‐prey detections by microscopy.

Pacific herring and walleye pollock were the only prey species confirmed by both methods, each in a different sample from Juan de Fuca Strait. However, DNA methods detected herring in many more samples than did microscopy, likely because the hard parts had been digested beyond visual recognition (e.g., DNA barcoding confirmed herring from “unidentified” bone fragments). Conversely, microscopy recovered anchovy bones in a sample, though anchovy was not detected by DNA metabarcoding. In this case, the proportion of anchovy in the whale's digestive tract might also have been low, relative to the herring prey that strongly amplified in this sample (sample O). Also, although walleye pollock, Pacific hake, sablefish, and Pacific herring had very high read abundances in one sample each, only pollock and herring bones were recovered. Northern anchovy bones were the only other bones recovered in this study, mixed in with herring bones in the sample with the very high herring DNA reads (sample O).

## 4 DISCUSSION

We describe fine‐scale diversity of fish and marine invertebrate prey detected in 14 samples of humpback whale feces obtained from three regions in southern BC. Our results suggest that Pacific herring may be an important prey species for humpback whales that forage in Juan de Fuca Strait and the Strait of Georgia, based on high frequency of detection, very high herring DNA read counts in three samples, and herring bones in two samples collected from these two regions. Invertebrate prey species inferred by DNA metabarcoding were predominantly from euphausiids (krill), with *Euphausia pacifica* being the most abundant and prevalent invertebrate inferred by DNA from all regions in this study. However, shrimp may also be important invertebrate prey in the Strait of Georgia and Juan de Fuca Strait, given that DNA from sergestid shrimp was detected in three samples, while mysid shrimp remains were recovered in seven samples from these regions.

The frequency of occurrence of herring and euphausiid DNA detections in the feces is consistent with surface prey observation data for humpback whales feeding in BC waters (Fisheries and Oceans Canada, 2013). However, we also recovered high read abundances from walleye pollock and Pacific hake in one sample each from Juan de Fuca Strait, as well as high‐read counts from sablefish in one sample from the western entrance to Juan de Fuca Strait, suggesting that these semi‐pelagic species may occasionally be targeted if juvenile size classes are encountered in sufficient densities. In general, we found that samples obtained from inside Juan de Fuca Strait contained the greatest diversity of prey species, particularly for fish, although substantially more samples were collected from this region, likely increasing the ability to detect prey diversity in the samples.

Food digestion in animals is a function of many variables, including an individual's activity level and the size and efficiency of its intestinal tract (Markussen, 1993). We observed heavily digested fecal material that drove substantial dissimilarities in taxonomic resolution between PCR‐based and morphological analyses of the feces. Our visual survey of the fecal samples recovered bones from just three species of fish (herring, anchovy, and pollock), whereas DNA detected the presence of 11 fish species. Notably, two fecal samples had large numbers of sequences from Pacific hake and sablefish, yet only a single rib bone was detected in the sablefish sample, and no bones were detected in the hake sample.

One possible explanation for the discordance between fish DNA and bones in this study is that digestive fluids are acidic and the skeletons of fish prey may have differentially eroded (Jobling & Breiby, 1986). For instance, walleye pollock bones may be more resistant to digestive acids than the more fragile bones of salmon and other species (Tollit et al., 2017), which may rapidly erode and dissolve during digestion (Jobling & Breiby, 1986; Tollit et al., 2007, 2017). The species of fish and the animal's gut transition time are therefore relevant to the extent of bone erosion prior to the animal defecating (Tollit et al., 2007). Food transit time through the digestive system of captive harbor seals (*Phoca vitulina*), for example, is between 2 and 6 h (Markussen, 1993), and salmonid bones have been identified in free‐ranging harbor seal feces in BC and Washington State (Thomas et al., 2022), in which the fragile otoliths of juvenile salmonids are often severely eroded (Thomas et al., 2017). Compared to seals, digestion time in rorqual whales is estimated to be considerably longer, possibly 15–18 h in fin whales (Víkingsson, 1997), though inter‐ and intraspecific variability in food passage time in rorquals remains unknown. Nevertheless, the cetacean stomach is multichambered (in contrast with pinnipeds) and well adapted for breaking down chitinous prey (Horstmann, 2017). Skeletal elements recovered in our study may therefore represent species whose bones were robust enough to survive transiting the whale's digestive tract. Furthermore, we would expect skeletal remains in the feces of all rorquals, not just humpback whales, to also represent either a recent consumption of that prey (i.e., fast passage through the digestive tract), or an elevated feeding frequency on it (Tollit et al., 2017).

In our study, sequences annotating to the humpback were the most abundant with whale sequences accounting for an average of 88% of the 12S rRNA gene sequences and 98% of COI sequences. The presence of host DNA served as a useful tool for confirming that each sample was feces; four samples were removed from further analysis because the 12S rRNA gene data had no whale sequence data. Given that collectors without a scientific research license needed to wait for the whale to move away from the area before collecting the fecal sample, the signal of whale DNA was a good test for whether or not the sample contained feces. However, the high frequency of whale sequences may also have obscured the presence of additional rare prey items. Additional sequencing could help to uncover the presence of rare prey items, but we suggest that the development of host‐specific blocking primers would be an improvement on the current study for uncovering additional prey species.

A predator's meal size, prey digestibility, and size of prey may also affect the number of sequence reads recovered from the feces (Deagle et al., 2010; Pompanon et al., 2012). For this reason, the relative number of sequence reads in the fecal samples belonging to any given prey species is not necessarily correlated with its abundance in the diet (Deagle et al., 2019). For example, samples with a high proportion of sequences recovered from a single prey item (e.g., walleye pollock) may only indicate a very recent consumption of that fish (Deagle et al., 2005). Given the limitations of sequence abundance, we suggest that ‘prevalence’—the proportion of fecal samples that contained a certain prey item—is a good metric for identifying prey species that may be preferentially targeted by humpback whales. Based on prevalence, fish including Pacific herring, Pacific hake, and eulachon were the most common fish species potentially targeted by whales in this study. Similarly, the krill *E. Pacifica* was the single most prevalent prey species encountered—being present in all but one fecal sample.

### 4.1 Local foraging ecology

North Pacific humpback whales consume a variety of regionally available small‐bodied prey from multiple trophic levels (Fleming et al., 2016; Witteveen et al., 2011). In our study, DNA metabarcoding inferred the presence of ecologically diverse fishes including anadromous species (salmon), pelagic species (pollock, hake, sablefish), and species inhabiting nearshore rocky areas (sculpins). We inferred the presence of Chinook (five samples) and coho salmon (four samples) in the fecal samples collected from inside Juan de Fuca Strait and the Strait of Georgia. The frequency of salmon detections from these two regions may indicate a relevant, albeit occasional, prey item in these areas. Fish such as pelagic hake and salmonids are not thought to be common humpback whale prey, as they do not generally aggregate at sufficiently high densities to compensate for the energetically demanding lunge feeding tactics of humpback whales (Chenoweth et al., 2017; Goldbogen et al., 2008). However, humpback whales are regularly observed foraging among the transect lines during Fisheries and Ocean Canada (DFO) integrated Pacific hake surveys in BC waters when hake and euphausiid densities are high (S. Gauthier. DFO, personal communication. February 2022). Additionally, a DFO winter trawl survey of juvenile coho salmon in southern BC captured 15,000 juvenile coho in an anomalous single tow over a 1‐h period (Beacham et al., 2016), indicating that young coho may at least sometimes school at densities of interest to foraging humpback whales.

Salmon hatcheries also are thought to provide annual prey subsidies to opportunistic marine predators in BC (Nelson et al., 2019). In southeast Alaska, individual humpback whales have learned to target hatchery‐release sites during releases of juvenile chum, Chinook and coho salmon (Chenoweth et al., 2017). Within the Salish Sea (Strait of Georgia, Juan de Fuca Strait, and Puget Sound), young hatchery salmon are released every year from April through June by numerous U.S. and Canadian public and private hatcheries (Kendall et al., 2020). Off southern Vancouver Island alone, more than 11 million Chinook and over 450,000 coho were released annually into the Strait of Georgia and Juan de Fuca Strait during our study period (RMIS database, www.rmpc.org). Humpback whales specialize in corralling small schooling fishes (Sharpe & Dill, 1997), and an opportunistic whale that encounters young salmon could herd them into denser schools for consumption (Chenoweth et al., 2017). Humpback whales and salmon of any age class (wild or hatchery) also may co‐occur in space and time and likely feed on the same herring school or krill swarm. Whether juvenile salmon would be targeted by humpbacks or swallowed incidentally is unknown; however, only smaller fish (≤30 cm) are likely to cycle through the whale's digestive track, and the targeted prey should therefore dominate the food DNA in the whale's fecal contents (Deagle et al., 2019).

### 4.2 Technical limitations

In all DNA metabarcoding studies, there is the potential for contamination by exogenous DNA during sample collection and handling. In our study, it was impossible to anticipate when a humpback whale would defecate, and sample collections were often done opportunistically using equipment that was available on the boat, such as a bucket or plastic container in some cases. It is possible that in these cases exogenous DNA fragments from the samplers' hands, the boat, or the collection method could potentially have indirectly contaminated the samples. Similarly, prior to transferring the archived samples to a molecular lab, the frozen samples had been opened in a university ecology lab, which may have contributed exogenous DNA fragments to the samples (Goldberg et al., 2016; Pompanon et al., 2012). We attempted to account for contamination during this initial sample processing step by sequencing a ‘lab control’ consisting of sterile water that was manipulated using an identical protocol as the fecal samples. However, it is possible that low‐level contamination may still have been present.

We conducted our analysis under the assumption that the relative abundance of DNA sequences from primary prey would dominate those generated from contaminants (Deagle et al., 2019). If DNA contamination had been a significant factor in our data, then we would have anticipated more consistent species detections across all samples, with contaminant species occurring in all samples. Instead, our fecal data were highly variable, and the abundant/prevalent prey items occurred at much greater abundance than in any of the controls. The high read abundances from specific species in single samples (e.g., pollock, hake, and sablefish) suggest that, if present, DNA from contaminants in the feces were negligible compared to DNA from prey consumed by the whale (de Vos et al., 2018; Deagle et al., 2019).

Free‐floating environmental DNA (eDNA) in seawater is another potential source of DNA contaminants in whale feces (de Vos et al., 2018). Aquatic organisms continuously shed DNA in mucus, skin, and feces (Lamy et al., 2021), to such an extent that previous studies have employed marine eDNA metabarcoding of water samples to describe the diversity of coastal fish communities based on eDNA (Afzali et al., 2021; Aglieri et al., 2021; Lamy et al., 2021). Whenever possible, we used a control seawater sample collected from the same area as each fecal sample in order to filter out background eDNA signals from our fecal samples. For example, salmon are highly mobile predators, and Chinook in particular may be nearly ubiquitous in the water column in BC coastal waters (W. Duguid, UVic, personal communication. March 2022), emphasizing the critical importance of control seawater samples in future DNA‐based rorqual diet studies. A further complexity is that fish are both predators and prey (Traugott et al., 2021). Many samples in our study contained a mix of low‐abundance species sequences (e.g., krill, copepod, and sculpin) that may simply reflect the stomach contents of fish consumed by the whale, and therefore secondary predation (Pompanon et al., 2012; Traugott et al., 2021).

## 5 CONCLUSIONS AND FUTURE DIRECTIONS

The energetic constraints of prey engulfment and filtration in rorquals require that the whales seek areas of high prey abundance, wherein they execute discrete lunge‐feeding events on patches of high‐density prey (Kahane‐Rapport et al., 2020; Potvin et al., 2021). Prey occurrences in humpback whale feces should therefore provide meaningful insight into the dominant taxa in food patches selected by the animal (Deagle et al., 2019). In this study, while the relatively high number of species detected in many samples suggests a diverse diet and generalist predation, a given sample likely contained DNA from multiple meals. Additionally, the dominance of semi‐pelagic fishes (that differed by species in a few samples in our study) supports the hypothesis that humpback whales may engage in selective subsurface foraging (Cade et al., 2022; Friedlaender et al., 2020; Witteveen et al., 2008, 2015). We assumed that all four sampling years (2017–2020) were similar oceanographically (i.e., all warm years; Boldt et al., 2019, 2020, 2021). The potential for selective foraging behavior should be further explored using DNA techniques over multiple years and across regions to gain better insight into the relative importance of particular prey species for this rapidly growing humpback whale population. In general, we found that molecular and visual analyses of fecal samples provide a complementary approach to diet analysis, with each method providing unique insight into prey diversity.

## AUTHOR CONTRIBUTIONS

**Rhonda D. Reidy:** Conceptualization (lead); data curation (lead); formal analysis (lead); funding acquisition (supporting); investigation (lead); methodology (equal); project administration (equal); resources (equal); supervision (equal); visualization (lead); writing – original draft (lead); writing – review and editing (lead). **Matthew A. Lemay:** Conceptualization (supporting); data curation (lead); formal analysis (lead); funding acquisition (supporting); investigation (supporting); methodology (lead); project administration (supporting); resources (supporting); supervision (supporting); visualization (supporting); writing – original draft (supporting); writing – review and editing (supporting). **Katie G. Innes:** Data curation (supporting); investigation (supporting); methodology (lead); writing – review and editing (supporting). **Rute B. G. Clemente‐Carvalho:** Data curation (supporting); methodology (lead); writing – review and editing (supporting). **Carly Janusson:** Methodology (supporting); writing – review and editing (supporting). **John F. Dower:** Writing – review and editing (supporting). **Laura L. E. Cowen:** Funding acquisition (supporting); supervision (supporting); writing – review and editing (supporting). **Francis Juanes:** Funding acquisition (supporting); project administration (supporting); resources (supporting); supervision (supporting); writing – review and editing (supporting).

## CONFLICT OF INTEREST

The authors have declared no conflict of interest.

## FUNDING INFORMATION

Funding was provided to RR was supported through a MITACS Accelerate fellowship in partnership with the PWWA and Shaw Centre for the Salish Sea, and the Eugene Maughan Scholarship, Western Division of the American Fisheries Society. FJ, LC were funded through grants from the NSERC Discovery program and FJ through the Liber Ero Foundation. Funding for the genetic analyses was provided by the Tula Foundation. Close whale approaches were done under DFO permit MML‐45 and institutional AUC protocols.

## Supporting information


Appendix S1

## Data Availability

Raw Illumina sequence data have been deposited at the NCBI Sequence Read Archive (BioProject ID = PRJNA859910).
