# Supplementary material for: Fine‐scale diversity of prey detected in humpback whale feces
Source: Ecol Evol. 2022 Dec 28;12(12):e9680. doi: 10.1002/ece3.9680 (PMC9797768; doi:10.1002/ece3.9680)
Supplement: Supplementary file 1 — Appendix S1 [file ECE3-12-e9680-s001.docx]

**Appendix Table A.** Relative proportion of fish and invertebrate sequences present in each 20mL control seawater sample. Control samples were only collected for six whale samples; the number of reads of each species present in the seawater control was subtracted from the corresponding faecal sample. Letters used for the sample names correspond to the faecal sample IDs.

|  | **Juan de Fuca Strait** | |  |  | **Swiftshure Bank** | |
| --- | --- | --- | --- | --- | --- | --- |
| **Invertebrates (COI)** | **B2** | **C2** | **K2** | **O2** | **Q2** | **R2** |
| Acartia longiremis | 1.000 |  | 0.789 |  |  |  |
| Unidentified copepods |  |  | 0.300 |  |  |  |
| Euphausia pacifica |  |  |  | 1.000 |  |  |
| Bivalve (Family: Veneridae) |  | 1.000 | 0.158 |  |  |  |
|  |  |  |  |  |  |  |
| **Fish (12S)** | **B2** | **C2** | **K2** | **O2** | **Q2** | **R2** |
| Pacific herring | 0.340 | 0.787 | 0.423 | 0.463 | 0.518 |  |
| Pink salmon | 0.238 | 0.486 |  | 0.232 | 0.388 |  |
| Atlantic salmon | 0.960 | 0.145 |  |  |  |  |
| High cockscomb |  | 0.882 |  | 0.976 |  |  |
| Rockfish |  |  |  |  | 0.525 | 0.548 |
| Scalyhead sculpin |  |  |  | 0.420 |  | 0.714 |
| Bluntnose sixgill shark |  |  | 0.155 |  |  |  |
| Buffalo sculpin |  |  |  |  | 0.926 |  |
| Butter sole |  |  |  |  |  | 0.294 |
| C-O sole |  |  |  |  |  | 0.132 |
| Cabezon |  |  |  | 0.120 |  |  |
| Chinook salmon |  | 0.762 |  |  |  |  |
| Chum salmon |  | 0.354 |  |  |  |  |
| Coho salmon | 0.546 |  |  |  |  |  |
| Kelp greenling |  |  |  |  | 0.834 |  |
| Lingcod |  |  |  |  | 0.127 |  |
| Pacific hake |  |  | 0.554 |  |  |  |
| Pacific grenadier |  |  |  |  |  | 0.364 |
| Pacific sanddab |  | 0.189 |  |  |  |  |
| Pacific sandfish |  |  |  |  |  | 0.122 |
| Pacific sandlance |  |  |  |  | 0.333 |  |
| Pacific staghorn sculpin |  |  |  | 0.569 |  |  |
| Prickly sculpin |  |  | 0.367 |  |  |  |
| Rock sole |  | 0.182 |  |  |  |  |
| Roughback sculpin | 0.784 |  |  |  |  |  |
| Shiner perch |  |  |  | 0.231 |  |  |
| Slender blacksmelt |  |  |  | 0.249 |  |  |
| Sockeye salmon |  | 0.586 |  |  |  |  |
| Speckled sanddab |  |  |  | 0.136 |  |  |
| unknown greenling |  |  |  |  | 0.158 |  |
| Wolf eel |  |  |  |  |  | 0.528 |
| Padded sculpin |  |  |  |  | <.1 |  |
| Penpoint gunnel |  |  |  |  | <.1 |  |
| Slimy sculpin |  |  |  |  | <.1 |  |

**Appendix Table B.** Relative proportion of fish prey taxa and humpback whale inferred from amplicon sequencing the 12S rRNA (MiFish) gene region.

|  | **Juan de Fuca Strait** | | | | | | | | | **Georgia Strait** | | **Swiftshure Bank** | |  |
| --- | --- | --- | --- | --- | --- | --- | --- | --- | --- | --- | --- | --- | --- | --- |
| **Common Name** | **A** | **B** | **C** | **G** | **K** | **M** | **N** | **O** | **P** | **E** | **J** | **Q** | **R** | **S** |
| Humpback whale | 0.9995 | 0.9992 | 0.9968 | 0.9968 | 0.1587 | 0.6112 | 0.9992 | 0.7287 | 0.9998 | 0.9115 | 0.9993 | 0.9997 | 0.9999 | 0.8178 |
| Pacific herring |  |  | 0.0008 | 0.0024 | 0.1097 |  |  | 0.2712 |  | 0.0786 |  | 0.0003 |  | 0.0029 |
| Pacific Hake | 0.0003 | 0.0003 |  | <0.0001 | 0.2611 |  |  | <0.0001 |  | 0.0003 | 0.0002 |  |  |  |
| Chinook salmon |  | 0.0001 |  | 0.0004 | 0.0426 |  | 0.0005 |  |  | 0.0068 |  |  |  |  |
| Eulachon |  | 0.0001 | 0.0002 | <0.0001 |  | 0.0001 |  | 0.0001 | <0.0001 |  |  |  |  |  |
| Coho salmon |  |  | 0.0004 | 0.0001 | 0.1017 |  |  |  |  | 0.0013 |  |  |  |  |
| Scalyhead sculpin |  |  |  | <0.0001 |  |  | 0.0001 |  | 0.0001 |  | 0.0003 |  |  |  |
| Rockfish (Sebastes sp.) |  | 0.0002 |  | <0.0001 |  |  |  |  | <0.0001 | 0.0007 |  |  | 0.0001 |  |
| Plain sculpin |  |  | 0.0003 |  |  |  | 0.0001 |  |  |  |  |  |  |  |
| High cockscomb |  | 0.0001 |  |  |  |  | <0.0001 |  | <0.0001 | 0.0003 |  |  |  |  |
| California headlightfish |  |  | <0.0001 | 0.0001 |  | <0.0001 | 0.0001 |  |  |  |  |  |  |  |
| Three-spined stickleback |  |  |  |  | 0.0560 |  |  |  |  | 0.0002 |  |  |  |  |
| Shiner perch | 0.0002 |  | 0.0007 |  |  |  |  |  |  |  |  |  |  |  |
| Tube-snout |  |  |  |  |  |  |  |  |  | 0.0001 |  |  |  | 0.0001 |
| Penpoint gunnel |  | 0.0001 |  |  | 0.0001 |  |  |  |  |  |  |  |  |  |
| Alaska pollock |  |  |  |  |  | 0.3887 |  |  |  |  |  |  |  |  |
| Chum salmon |  |  |  |  | 0.0407 |  |  |  |  |  |  |  |  |  |
| Sockeye salmon |  |  |  |  | 0.0117 |  |  |  |  |  |  |  |  |  |
| Pink salmon |  |  |  |  | 0.0081 |  |  |  |  |  |  |  |  |  |
| Slimy sculpin |  |  | <0.0001 |  | 0.1493 |  |  |  |  |  |  |  |  |  |
| Red Irish lord |  |  | 0.0004 |  |  |  |  | <0.0001 |  |  |  |  |  |  |
| Padded sculpin |  |  |  |  |  |  |  |  |  |  |  | <0.0001 |  | 0.0001 |
| Buffalo sculpin |  |  |  |  |  |  |  |  |  | <0.0001 | 0.0001 |  |  |  |
| Prickly sculpin |  |  |  |  | 0.0601 |  |  |  |  |  |  |  |  |  |
| Cabezon |  |  |  |  | 0.0002 |  |  |  |  |  |  |  |  |  |
| Sablefish |  |  |  |  | <0.0001 |  |  |  |  |  |  |  |  | 0.1788 |
| Striped seaperch |  |  |  |  |  |  |  |  |  | 0.0001 |  | <0.0001 |  |  |
| Ribbon prickleback |  |  |  |  |  |  |  |  | <0.0001 |  | 0.0001 |  |  |  |
| Butter sole |  |  |  |  |  |  |  |  |  |  |  |  |  | 0.0002 |
| Pacific grenadier |  |  | 0.0002 |  |  |  |  |  |  |  |  |  |  |  |
| Kelp perch |  |  |  |  |  |  |  | 0.0001 |  |  |  |  |  |  |
| Pacific sandlance |  |  | <0.0001 |  |  |  |  |  |  |  |  |  |  |  |

**Appendix Table C.** Relative proportion of invertebrate prey taxa and humpback whale inferred from amplicon sequencing a ~313bp fragment of the COI gene region.

|  | **Juan de Fuca Strait** | | |  |  |  |  |  |  | **Georgia Strait** | | **Swiftshure Bank** | |  |
| --- | --- | --- | --- | --- | --- | --- | --- | --- | --- | --- | --- | --- | --- | --- |
| **Species** | **A** | **B** | **C** | **G** | **K** | **M** | **N** | **O** | **P** | **E** | **J** | **Q** | **R** | **S** |
| Humpback Whale | 0.9861 | 0.9977 | 0.9976 | 0.9904 | 0.9383 | 0.9996 | 0.9986 | 0.9873 | 0.9988 | 0.9550 | 0.9418 | 0.9999 | 0.9977 | 0.9888 |
| Euphausia pacifica | 0.0105 | 0.0013 | 0.0016 | 0.0066 |  | 0.0003 | 0.0009 | 0.0127 | 0.0011 | 0.0450 | 0.0579 | 0.0001 | 0.0022 | 0.0098 |
| Thysanoessa spinifera | 0.0014 | 0.0001 | 0.0001 | 0.0003 |  |  | 0.0001 |  | 0.0001 |  | 0.0002 |  | 0.0001 | 0.0001 |
| Thysanoessa raschii | 0.0017 | 0.0009 | 0.0007 | 0.0012 |  | 0.0001 | 0.0003 |  |  |  |  |  |  |  |
| Sergestes similis | 0.0003 |  |  | 0.0014 |  |  | 0.0001 |  |  |  |  |  |  |  |
| Eualus avinus |  |  | <0.0001 | 0.0002 |  |  |  |  |  |  |  |  | <0.0001 |  |
| Acartia longiremis |  |  |  |  |  |  |  |  |  |  |  |  |  | 0.0009 |
| Calanus pacificus |  |  |  |  | 0.0532 |  |  |  |  |  |  |  |  |  |
| Ditrichocorycaeus anglicus |  |  |  |  |  |  |  |  |  |  | 0.0001 |  |  |  |
| Haminoea vesicula |  |  |  |  | 0.0085 |  |  |  |  |  |  |  |  |  |
| Anisakis simplex |  |  |  |  |  |  |  |  |  |  |  |  |  | 0.0002 |
| Platynereis bicanaliculata |  |  |  |  |  |  |  |  |  |  |  |  |  | 0.0002 |
